# Therapeutic and Diagnostic Innovation for Parasitology: Grand Challenges

**DOI:** 10.3389/fpara.2022.963671

**Published:** 2022-07-04

**Authors:** Richard J. Martin

**Affiliations:** Department of Biomedical Sciences, Iowa State University, Ames, IA, United States

**Keywords:** therapeutics, diagnostics, vaccination, parasitology, resistance, challenges

## Background

Parasites exact a very heavy burden on the health of human and animal populations. In humans this problem is more severe in developing countries (Pullan and Brooker, 2008; Hotez et al., 2014). In animals, parasites affect productivity and raise welfare issues. In humans the parasites include: protozoa like *Plasmodium falciparum* that cause malaria; filarial nematode parasites like *Onchocerca volvulus* that cause river blindness that are transmitted by biting insects; soil-transmitted helminths like *Ascaris lumbricoides, Trichuris trichiura* and Ancylostoma duodenale and Necator americanus; and a variety of insect ectoparasites (https://www.medicalnewstoday.com/articles/human-parasites#types-of-parasites). Similar parasite species are seen in animals where they have a negative impact on animal health, welfare and food production (Fitzpatrick, 2013). Many life cycle stages of parasites are impacted by the environment during transmission or development and there is a real concern about how the climate will affect parasite populations as it continues to warm (Gehman et al., 2018). Given the massive and world-wide health problems that parasitic diseases produce and the increasing movement of human populations from developing countries to developed countries, it is imperative for our future wellbeing that we advance techniques for the rapid diagnosis and treatments of the many parasitic diseases.

## The Different Groups of Parasites Require Different Drugs for Treatments

**Figure 1:**
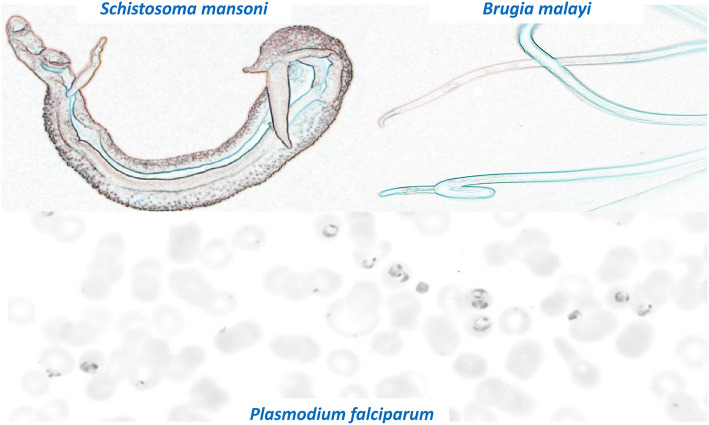
Colorized images of three significant and diverse parasites that are part of the Grand Challenges for Therapeutic and Diagnostic Innovation for Parasitology. Note the very different appearances and sizes of the different parasites. Top left: Male and Female *S. mansoni* (length 10 mm). Top Right: *B. malayi* (up to 45 mm length). Bottom panel: *P. falciparum* within RBCs (Size around 10 μM).

One of the problems facing parasitologists is the enormous number and diverse biology of the parasites that require investigation which reduces research focus and slows progress. Figure 1 shows some colorized images of three very diverse parasites that are part of the Grand Challenges for Therapeutic and Diagnostic Innovation for Parasitology. These parasites range from unicellular protozoa to multicellular helminths and ectoparasites and have much larger genomes (up to 370 Mb) than bacteria and viruses. The size of parasite genomes approaches that of humans that have a genome size of some 700 Mb. The large genome of some parasites allows increased ability of these parasites to adapt to change and to develop resistance to xenobiotic drugs. We need to discover and understand the mechanisms of resistance that parasites develop to drug treatment and to limit its effect by innovative drug design and early detection of resistance with new diagnostic techniques.

The vastly different metabolisms and biochemistry of protozoa and helminth parasites means that treatments are usually disease specific and sometimes limited to the life-cycle stage of the parasites. The drug praziquantel used for the treatment of schistosomiasis can be effective against *Schistosoma mansoni* adult parasites but is less effective against juvenile stages of the parasite (Vale et al., 2017). Praziquantel is not effective for the treatment of roundworm helminth infections. Pyrantel is effective against ascariasis, but is not effective against trichuriasis, both soil-transmitted helminths: as a result, oxantel had to be developed for trichuriasis (Hansen et al., 2021). The drugs used for the treatment of malaria, including dihydroartemisinin and piperaquine, are not effective against helminths. The drugs used for the treatments of helminths, albendazole and ivermectin are not used for the treatment of protozoan diseases. There is a need to develop improved therapeutic agents for many of the parasitic diseases.

## Resistance Is a Major Concern, Requiring Innovation, and Development of Resistance Busting Drugs

There is a wide range of different parasitic diseases that require new and effective drugs and tests for resistance. There are only three main classes of anthelmintic drug to treat nematode helminths, the benzimidazoles (albendazole), the cholinergics (pyrantel) and the macrocyclic lactones (ivermectin). For the treatment of schistosomiasis there are few other options: treatment is effectively limited to praziquantel. For malaria, artemisinin-based combination therapies are often used. For Chagas disease, a protozoan infection benznidazole or nifurtimox are used. The concern is that the limited number of classes of drugs that are used for treatment and control, has and will, continue to give rise to the development of resistance. There is then an urgent need for innovation and development of novel resistance-busting therapeutic drug classes and for early tests that detect the emergence of resistance.

## Vaccines are Required

The regular use of a limited number of antiparasitic drugs to control infections has given rise to the development of drug resistance in malaria against chloroquine and is emerging against the artemisinins. Resistance is also seen in nematode helminths against the benzimidazoles in animals and is a concern for human infections for soil transmitted helminths. There is evidence of resistance in *S. mansoni* against praziquantel. The regular use of the limited number of drugs has contributed to the rise in resistance. Rather than relying on the continuous development of drugs for prevention, effective vaccines should be developed. Development of malaria vaccines (https://www.cdc.gov/malaria/malaria_worldwide/reduction/vaccine.html), hookworm vaccines (Chapman et al., 2021a,b) and schistosomiasis vaccines (https://www.kpwashingtonresearch.org/news-and-events/recent-news/news-2022/researchers-begin-clinical-trial-assess-schistosomiasis-vaccine) could make a very important contribution to the control of parasitic diseases. The ability of different parasites to evade the host immune system adds to the difficulty of developing effective vaccines. The efficacy of the vaccines, to be successful, may not have to be >90% because even a reduction in the parasite levels to 60% or more can interrupt transmission in low and moderate transmission settings and would have an important health benefit (Stylianou et al., 2017). Parasite vaccine innovation is now required.

## Diagnostic Test Innovation is Required

Diagnosis is the first and primary stage before successful treatment of clinical conditions. Although the history of an individual and a knowledge of the local disease prevalence will be a diagnostic guide, differential diagnosis requires improved diagnostic tests (Ospina-Villa et al., 2020). Even with malaria, it is important to differentiate between *P. falciparum* and *P. vivax* and the rarer *P. ovale* malariae. Other protozoan diseases including toxoplasmosis, Chagas disease, trypanosomiasis, leishmaniasis need improved diagnostic tests as well. Many helminth diseases including the soil-transmitted helminth infections with *Ascaris*, hookworm or whipworm use older fecal egg counting techniques for diagnosis that are slow and not always accurate. It is very desirable for more reliable, robust and modestly priced antigen tests to be developed. Innovation improving accuracy and ease of use for mass screening as well as individual diagnostic testing is required. Molecular tests that can detect and track the development of resistance are urgently needed but are sadly lacking.

## Reasons for Being Involved in the Research

The innovative research required for development of novel therapeutics and diagnostic tests has a very useful and necessary utilitarian value: to control and limit the spread of the dreadful diseases caused by parasites. The number of scientists involved in the research is smaller than other areas of biomedical research, partly because of the limited economic drivers for diseases in developing countries, and partly because the low visibility of these diseases in developed countries. For those that are involved in the research, they have a bigger opportunity to make a significant impact on the field. Another reason to be involved in the research is the fascinating biology of the different parasites, how they evade the host immune systems, how these more complex pathogens respond to exposure to xenobiotic drugs and adapt to survive. Scientific curiosity is also a major driver for researchers in this field. We have a real opportunity to advance the control of parasite infectious diseases and discover new biology.

## Author Contributions

The author confirms being the sole contributor of this work and has approved it for publication.

## Funding

This study was supported by NIH NIAID Grants R01AI047194, R01AI155413, and R21AI138967 to RM and the EA Benbrook Endowed Chair of Pathology and Parasitology. The funding agencies had no role in the design, execution, or publication of this study.

## Author Disclaimer

The content is solely the responsibility of the authors and does not necessarily represent the official views of the National Institute of Allergy and Infectious Diseases.

## Conflict of Interest

The author declares that the research was conducted in the absence of any commercial or financial relationships that could be construed as a potential conflict of interest.

## Publisher's Note

All claims expressed in this article are solely those of the authors and do not necessarily represent those of their affiliated organizations, or those of the publisher, the editors and the reviewers. Any product that may be evaluated in this article, or claim that may be made by its manufacturer, is not guaranteed or endorsed by the publisher.
